# Circ_RNF13 Regulates the Stemness and Chemosensitivity of Colorectal Cancer by Transcriptional Regulation of DDX27 Mediated by TRIM24 Stabilization

**DOI:** 10.3390/cancers14246218

**Published:** 2022-12-16

**Authors:** Yihang Guo, Gui Hu, Buning Tian, Min Ma, Fei Long, Miao Chen

**Affiliations:** Department of Gastrointestinal Surgery, The Third XiangYa Hospital, Central South University, Changsha 410013, China

**Keywords:** circ_RNF13, DDX27, TRIM24, colorectal cancer

## Abstract

**Simple Summary:**

Colorectal cancer (CRC) is associated with high incidence and poor prognosis globally. An expression profile has revealed that circ_RNF13 is upregulated in CRC tumor samples. The aim of this study is to unravel the biological function and regulatory mechanism of circ_RNF13 in CRC. Circ_RNF13 stabilized TRIM24 via suppressing FBXW7-mediated degradation, thereby upregulating DDX27 expression in a TRIM24-dependent manner. The circ_RNF13/TRIM24/DDX27 axis is implicated in the regulation of stemness and chemoresistance in CRC. These findings identified promising prognostic biomarkers and novel targets for CRC chemotherapy.

**Abstract:**

Background: Colorectal cancer (CRC) is one of the most commonly diagnosed cancers with high incidence and poor prognosis worldwide. Circ_RNF13 is upregulated in CRC; however, the biological roles and downstream signaling of circ_RNF13 remain undefined. Methods: The characterization of circ_RNF13 was determined by Sanger sequencing, qRT-PCR, subcellular fractionation assay, and RNA FISH. Western blot analysis and qRT-PCR were employed to detect the expression of the key molecules and stemness markers in CRC tumor samples and cells. The stem-like activities of CRC cells were assessed by sphere formation assay, flow cytometry, and immunofluorescence (IF). Cell viability was monitored by CCK-8 assay. The chemosensitivity of CRC cells was assessed by colony formation and cell apoptosis assays. Bioinformatics analysis, RIP assay, RNA pull-down assay, and FISH/IF staining were used to detect the association between circ_RNF13 and TRIM24. The transcriptional regulation of DDX27 was investigated by ChIP assay, and the post-translational regulation of TRIM24 was detected by Co-IP. The in vitro findings were verified in a xenograft model. Results: circ_RNF13 and DDX27 were elevated in CRC tumor samples and cells. Knockdown of circ_RNF13 or DDX27 inhibited stemness and increased chemosensitivity in CRC cells. Mechanistically, circ_RNF13 regulated DDX27 expression via TRIM24-mediated transcriptional regulation, and circ_RNF13 stabilized TRIM24 via suppressing FBXW7-mediated TRIM24 degradation. In vivo studies revealed that the knockdown of circ_RNF13 impaired stemness and enhanced the chemosensitivity of CRC in the xenograft model. Conclusion: circ_RNF13 regulated the stemness and chemosensitivity of CRC by transcriptional regulation of DDX27 mediated by TRIM24 stabilization.

## 1. Introduction

Colorectal cancer (CRC) with increasing incidence among younger adults remains the second leading cause of cancer death globally [1,2]. In 2020, there were over 900,000 CRC-related death worldwide [3]. The unfavorable prognosis and overall survival (OS) of CRC are mainly attributed to chemoresistance and distant metastasis [4]. Accumulating evidence illustrates that cancer stem cells (CSCs) play a vital role in cancer growth, metastasis, and chemoresistance [5]. There is an urgent need to identify the prognostic biomarkers and elucidate the underlying mechanism to overcome stemness and chemoresistance in CRC.

DDX27 belongs to the dead-box RNA helicase family [6]. It is required for ribosomal RNA (rRNA) maturation, as well as skeletal muscle growth and regeneration [7]. More importantly, DDX27 is highly amplified and overexpressed in CRC tumor samples [8,9]. Previous studies have demonstrated an oncogenic role of DDX27 in CRC. DDX27 regulates cell proliferation, metastasis, stemness, and chemosensitivity to 5-fluorouracil (5-FU) in CRC [8,9]. The GEO database shows that DDX27 is elevated in stem-like CRC cells, further indicating the important role of DDX27 in CRC stemness. However, the mechanism underlying DDX27-regulated stemness and chemoresistance in CRC remains largely uninvestigated.

circRNAs are a novel class of single-stranded RNA with a closed circular structure and are devoid of the 3′ poly(A) tails or 5′ caps [10]. In the past decade, circRNAs have emerged as key players in various human disease, including cancer [11,12]. Circ_RNF13, also known as hsa_circ_0001346, is dysregulated in different types of cancer. For instance, circ_RNF13 is downregulated in lung adenocarcinoma (LAD) and nasopharyngeal carcinoma (NPC), but it is upregulated in acute myeloid leukemia (AML) and pancreatic cancer (PC) [13,14,15,16]. Circ_RNF13 functions as either an oncogene or a tumor suppressor in different tumors [13,14,15,16]. An expression profile has illustrated that circ_RNF13 is elevated in CRC tumor samples, compared with its normal counterparts [17]. It is of interest to unravel the biological function and regulatory mechanism of circ_RNF13 in CRC. 

Tripartite motif containing 24 (TRIM24) is involved in the epigenetic regulation of transcription by nuclear receptors [18]. In addition, TRIM24 has been identified as an interacting partner of p53, and it is implicated in the ubiquitin-dependent degradation of p53 [19]. More importantly, a previous study has demonstrated the upregulation of TRIM24 in CRC tumor samples, and it is associated with several clinical parameters, such as tumor size, stage, and poor prognosis [20]. The RPISeq database predicted that there is a putative association between circ_RNF13 and TRIM24, and the binding sites between TRIM24 and the DDX27 promoter were predicted using AnimalTFDB. Bioinformatics analysis also identified FBXW7 as an E3 ubiquitin ligase responsible for TRIM24 degradation. This thus promotes us to hypothesize that circ_RNF13 might stabilize TRIM24 via suppressing FBXW7-mediated degradation, thereby upregulating DDX27 expression in a TRIM24-dependent manner. The circ_RNF13/TRIM24/DDX27 axis might be implicated in the regulation of stemness and chemoresistance in CRC.

In this study, we demonstrated that circ_RNF13 and DDX27 were upregulated in CRC tumor samples and cells. Silencing of circ_RNF13 or DDX27 inhibited stemness and increased chemosensitivity in CRC cells. Mechanistically, circ_RNF13 regulated DDX27 expression via TRIM24-mediated transcriptional regulation, and circ_RNF13 stabilized TRIM24 via suppressing FBXW7-mediated TRIM24 degradation. In vivo studies revealed that knockdown of circ_RNF13 impaired the stemness and enhanced the chemosensitivity of CRC in a xenograft model.

## 2. Materials and Methods

### 2.1. Collection of Clinical Specimens

The CRC tumor samples (n = 50) and their normal counterparts (n = 50) were collected from CRC patients at The Third XiangYa Hospital of Central South University. A cohort of 50 CRC patients (low circ_RNF13 n = 25; high circ_RNF13 n = 25) were recruited. This study was approved by The Third XiangYa Hospital of Central South University (No. 2021-S094). Written consent was obtained from all of the participants.

### 2.2. Cell Culture, Treatment, and Transfection

Normal colon cell line NCM-460 cells and human CRC cell lines CoLo205, DLD-1, HT-29, CoLo320, RKO, NCI-H716, and Caco-2 cells were from ATCC (Manassas, VA, USA). The NCM-460 cells were grown in M3 medium containing 10% FBS (Gibco, Grand Island, NY, USA). All of the CRC cell lines were grown in RPMI1640 supplemented with 10% FBS (Gibco) 37 °C/5% CO_2_. For oxaliplatin (L-OHP) treatment, HT-29 and Caco-2 cells were treated with different doses of L-OHP (0, 5, 10, 20 and 40 μg/mL). For irinotecan (CPT-11) treatment, HT-29 and Caco-2 cells were treated with different doses of CPT-11 (0, 5, 10, 20 and 40 μg/mL). To study the protein stability, cycloheximide (CHX, 20 μg/mL) was administered for 0.25, 0.5, 1, 2, and 4 h. To block the ubiquitin–proteasome pathway, the cells were treated with 20 μM MG132. CHX and MG132 were purchased from MedChemExpress. shNC, sh-circ_RNF13-1, sh-circ_RNF13-2, sh-DDX27-1, sh-DDX-27-2, sh-TRIM24, sh-FBXW7, sh-MDM2, sh-TRIM25, sh-NEDD4, sh-TRIM11, sh-RNF180, sh-SYVN1, sh-MIB1, and pcDNA3.1-circ_RNF13 were purchased from GenePharma (Shanghai, China). HT-29 and Caco-2 cells were transfected 0.5 μg shRNA and/or plasmids using Lipofectamine 3000 (Invitrogen, Carlsbad, CA, USA). For double knockdown, 0.5 μg sh-circ_RNF13-1 and/or 0.5 μg sh-FBXW7 were co-transfected into CRC cells. At 48 h post-transfection, cells were harvested for subsequent analysis.

### 2.3. qRT-PCR

Total RNA was isolated using TRIzol (Invitrogen) and subjected to reverse transcription using PrimeScript RT Reagent (TaKaRa, Dalian, China). qRT-PCR was conducted using SYBR Green PCR MasterMix (ABI, Foster City, CA, USA). The fold change was calculated using the 2^–ΔΔCT^ method. For the RNA stability assay, the cells were incubated with RNase R (20 U/μL, Epicenter, WI, USA) for 0, 10, 20, 30, and 40 min. The cells were treated with Actinomycin D (2 mg/mL, Sigma-Aldrich) for 0, 12, and 24 h. The expression of circ_RNF13 or RNF13 were detected by qRT-PCR. The divergent and convergent primers were designed as described [21]. The divergent primers were designed to span the back splicing sequence of circ_RNF13. The primers which amplify the region surrounding the back splicing junction with convergent orientation were designed for linear RNF13. The primers used in the qRT-PCR were listed in Table 1.

### 2.4. Western Blot Analysis

Protein lysates from CRC tumor samples and cells were prepared with RIPA lysis buffer (Beyotime, Nanjing, China). After gel electrophoresis, the proteins were transferred onto the PVDF membrane. The blot was then incubated with a primary antibody, followed by incubation with secondary antibody HRP. The protein bands were detected using ECL substrate (Pierce, Rockford, IL, USA), and the image was collected under the gel imaging system. ImageJ software was used to analyze the gray value of the protein band and calculate the protein expression. β-tubulin or GAPDH were used as a control. The antibodies used in Western blot analysis are listed in Table 2.

### 2.5. Fluorescence In Situ Hybridization (FISH) and Immunofluorescence (IF)

Cy3-labeled circ_RNF13 probes were synthesized by RiboBio (Guangzhou, China). The cells were fixed with 4% PFA and permeabilized. RNA FISH was performed using the FISH Kit (RiboBio). For IF staining, the cells were incubated with an antibody against TRIM24, followed by the incubation with secondary antibody Alexa Fluor 647. The co-localization of circ_RNF13 and TRIM24 were detected using a Nikon confocal microscope (Nikon, Tokyo, Japan) and analyzed using Manders’ overlap coefficient (MOC) as described [22,23]. An MOC > 0.6 was considered as a significant colocalization. 

### 2.6. Subcellular Fractionation

The nuclear and cytoplasmic fractions were separated using a PARIS kit (Invitrogen). Briefly, HT-29 and Caco-2 cells were lysed with cell fractionation buffer. After centrifugation, the supernatants were collected and designated as cytoplasmic lysates. The pellets were lysed with cell disruption buffer and designated as nuclear lysates. RNA isolation and qRT-PCR analysis were then conducted. 

### 2.7. Cell Counting Kit-8 (CCK-8) Assay

In brief, HT-29 and Caco-2 cells (5 × 10^3^) were plated into 96-well plates. After 4 h of treatment, 10 μL CCK-8 solution (DOJINDO, Kumamoto, Japan) was added into each well and incubated at 37 °C for 2 h. A450 was measured using a microplate reader (Thermo Fisher Scientific, Waltham, MA, USA).

### 2.8. Sphere Formation Assay

HT-29 and Caco-2 cells (1 × 10^3^) were replated into 6-well ultra-low attachment culture dishes and cultured in RPMI1640 containing 20 ng/mL b-FGF, 20 ng/mL EGF, 2% B27 supplement, and 4 mg/mL insulin (Gibco). After 14 days, the spheroid colonies (>50 μm in diameter) were photographed and counted.

### 2.9. Colony Formation Assay

The cells were plated into 60-mm dishes (0.5 × 10^3^ cells/plate). After 2 weeks, the crystal violet-stained colonies (>50 cells) were photographed and counted.

### 2.10. Cell Apoptosis Assay

In brief, HT-29 or Caco-2 cells were harvested and resuspended in binding buffer, followed by the staining with Annexin V-FITC and PI (Beyotime) for 15 min. The stained cells were then analyzed by a flow cytometer (BD, Franklin Lakes, NJ, USA).

### 2.11. Flow Cytometry

The cells were collected and digested with Accutase (Invitrogen). The cells were then stained with anti-CD133-APC and anti-CD44-FITC antibodies (Invitrogen). The stained cells were analyzed by a flow cytometer (BD). 

### 2.12. RNA Pull-Down Assay

The RNA pull-down assay was conducted using a Pierce RNA pull-down assay kit (Pierce). In brief, the biotinylated circ_RNF13 probe was conjugated to Streptavidin beads and incubated with cell lysates. The RNA–protein complexes were then eluted and subjected to Western blot analysis. 

### 2.13. RNA Immunoprecipitation (RIP) Assay

The RIP assay was conducted using a Magna RIP kit (Millipore, Billerica, MA, USA). In brief, anti-TRIM24 (2 μg) or normal IgG (2 μg)-conjugated beads were incubated with cell lysates at 4 °C overnight. The immunoprecipitated circ_RNF13 was purified and detected by qRT-PCR, and the successful IP was confirmed by Western blot analysis.

### 2.14. Chromatin Immunoprecipitation (ChIP) Assay

The ChIP assay was carried out using a Pierce Magnetic ChIP kit (Pierce). Briefly, the cells were crosslinked with 1% formaldehyde and subjected to MNase digestion. Anti-TRIM24 or normal IgG was conjugated to magnetic beads, followed by incubation with chromatin fractions. DNA purification and qRT-PCR were conducted to detect the immunoprecipitated DNAs. 

### 2.15. Co-Immunoprecipitation (Co-IP)

HT-29 and Caco-2 cells were lysed with IP lysis buffer (Pierce). After protein quantification, 1 mg cell lysates were then incubated with 2 μg anti-FBXW7, anti-TRIM24 antibody, or normal IgG at 4 °C overnight. The protein complexes were then enriched by Protein A/G beads (Pierce) at 4 °C for 4 h and subjected to Western blot analysis. Whole cell lysates and normal IgG served as an input and negative control, respectively. 

### 2.16. Animal Study

The male BALB/c nude mice (6-week-old, n = 6 per group) were from Hunan SJA Laboratory Animal Co., Ltd. (Changsha, China). Transfected HT-29 or Caco-2 cells with different cell densities were subcutaneously injected into the flank of the mice. The tumor size was monitored every 3 days and calculated with the formula: volume = 1/2 × length ×width^2^. On day 24, the tumors were harvested, weighed, and subjected to subsequent analysis. For L-OHP or CPT-11 treatment, mice with a xenograft tumor received intraperitoneal injection of 0.15 mg/kg L-OHP or 30 mg/kg CPT-11 twice per week for 4 weeks. All animal studies were approved by The Third XiangYa Hospital of Central South University (No. 2019sydw0235).

### 2.17. Immunohistochemistry (IHC)

After deparaffinization, rehydration, and antigen retrieval, the sections were incubated with an anti-TRIM24 or anti-DDX27 antibody. The slides were then incubated with secondary antibody HRP. The signals were detected using DAB substrate (Beyotime). 

### 2.18. TUNEL Assay

DNA fragmentation was assessed by using TUNEL Assay Kit-HRP-DAB (Abcam). In brief, fixed xenograft tumors were stained with staining solution at 37 °C for 1 h. The images were acquired using a microscope (Nikon).

### 2.19. Statistical Analysis

The data were presented as mean ± SD and were obtained from at least 3 independent experiments. One-way ANOVA or a Student’s *t*-test was conducted using GraphPad Prism software 8.0 (GraphPad, La Jolla, CA, USA). Survival curves were plotted and compared using the Kaplan–Meier method and the log-rank test, respectively, and the value of circ_RNF13 expression was obtained by median. *p* < 0.05 was considered statistically significant.

## 3. Results

### 3.1. Characterization of Circ_RNF13

**Figure 1:**
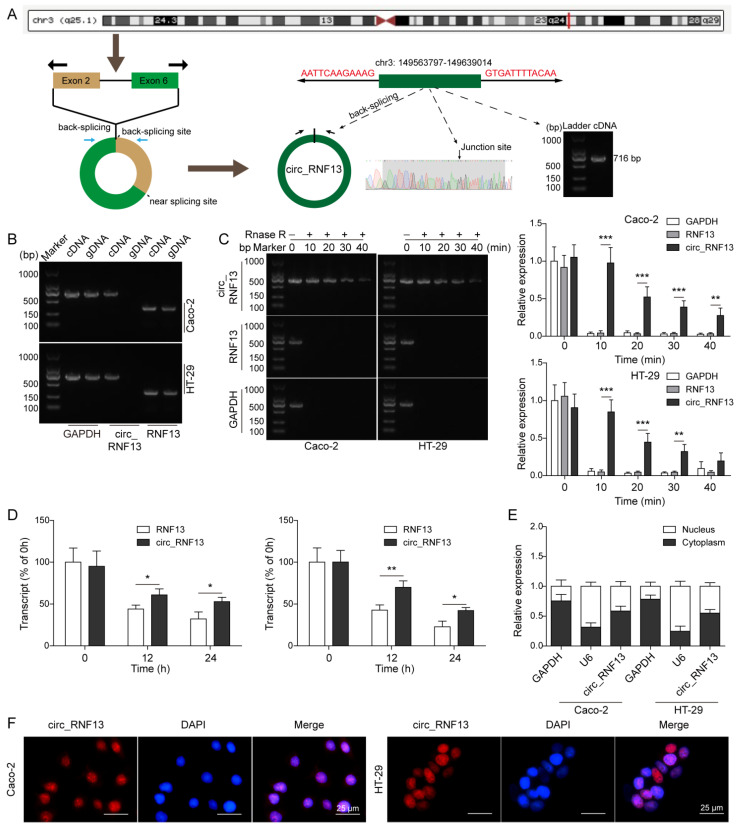
Characterization of circ_RNF13. (**A**) Schematic drawing of circ_RNF13 structure in the chromosome 3q25.1. (**B**) The amplifications of circ_RNF13 or linear RNF13 in cDNA and gDNA fractions were detected using different primers. (**C**) The stabilities of circ_RNF13 and RNF13 upon RNase R treatment were detected by qRT-PCR. (**D**) The stabilities of circ_RNF13 and RNF13 upon Actinomycin D treatment were detected by qRT-PCR. (**E**) The nuclear and cytoplasmic expression of circ_RNF13 were detected by subcellular fractionation assay. (**F**) The subcellular localization of circ_RNF13 in HT-29 and Caco-2 cells was assessed by FISH. Red, circ_RNF13; blue, DAPI. Scale bar, 25 µm. Each experiment was performed at least three times independently. *, *p* < 0.05, **, *p* < 0.01, ***, *p* < 0.001.

Sanger sequencing showed that circ_RNF13 was encoded in the chromosome 3q25.1, and it was derived from back-spliced exons 2–6 of the RNF13 gene. The full-length of circ_RNF13 is 716 bp (Figure 1A). To test that the amplification was from the circular template, different primers were used in qRT-PCR. As presented in Figure 1B, the divergent primers designed to amplify circ_RNF13 produced a single band with the expected size in the cDNA, but not in the genomic DNA (gDNA) fractions. On the other hand, the convergent primers for linear RNF13 produced bands in both cDNA and gDNA fractions, indicating the specificity of circ_RNF13 primers. In addition, the RNA stability assay revealed that circ_RNF13 degraded in a time-dependent manner upon RNase R or Actinomycin D treatment, and the degradation of circ_RNF13 was much slower than that of linear RNF13 (Figure 1C,D). Furthermore, the subcellular fractionation assay and FISH revealed that circ_RNF13 was expressed in both the nucleus and cytoplasm in HT-29 and Caco-2 cells (Figure 1E,F). These findings indicate the specificity of circ_RNF13 primers, as well as the nuclear and cytoplasmic expression of circ_RNF13 in CRC cells.

### 3.2. Circ_RNF13 and DDX27 Are Elevated in CRC Tumor Samples and Cells

**Figure 2:**
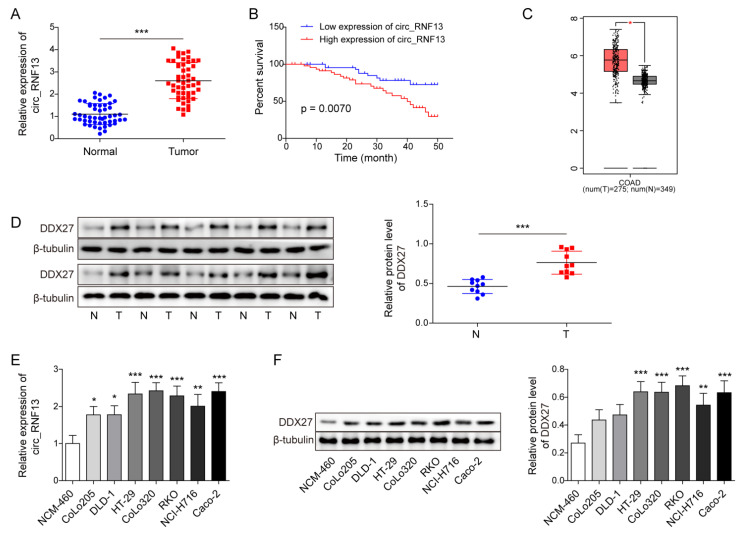
circ_RNF13 and DDX27 are upregulated in CRC tumor samples and cells. (**A**) The level of circ_RNF13 in CRC tumor samples was detected by qRT-PCR. (**B**) The correlation of circ_RNF13 level with OS of CRC patients. (**C**) Analysis of DDX27 expression in CRC tumor samples based on TCGA database. (**D**) The protein level of DDX27 in CRC tumor samples was detected by Western blot analysis. (**E**) The level of circ_RNF13 in CRC cells (CoLo205, DLD-1, HT-29, CoLo320, RKO, NCI-H716, and Caco-2) and normal colon cell line NCM-460 cells was detected by qRT-PCR. (**F**) The protein level of DDX27 in CRC cells (CoLo205, DLD-1, HT-29, CoLo320, RKO, NCI-H716, and Caco-2) and NCM460 was detected by Western blot analysis. Each experiment was performed at least three times independently. *, *p* < 0.05, **, *p* < 0.01, ***, *p* < 0.001. Full pictures of the Western blots are presented in Figure S2.

To study the roles of circ_RNF13 and DDX27 in CRC, we first examined their expression in CRC tumor samples. As shown in Figure 2A, circ_RNF13 was elevated in CRC tumor samples, compared with their normal counterparts. More importantly, Kaplan–Meier analysis revealed that patients with high circ_RNF13 levels exhibited poor OS, whereas low circ_RNF13 levels in CRC patients was associated with better OS (Figure 2B). In addition, circ_RNF13 was positively correlated with tumor size, distant metastasis, lymph metastasis, and TNM stage (Table 3). TCGA data revealed that DDX27 was upregulated by 2.33-folds in CRC tumor samples (Figure 2C). Consistent with this finding, Western blot analysis showed that DDX27 was induced approximately 1.65-fold in CRC tumor samples (n = 10) in comparison with the matched adjacent normal tissues (Figure 2D). Moreover, circ_RNF13 and DDX27 were upregulated in the CRC cells lines CoLo205, DLD-1, HT-29, CoLo320, RKO, NCI-H716, and Caco-2 cells compared with normal colon cell line NCM-460 cells (Figure 2E,F). HT-29 and Caco-2 cells with relatively high circ_RNF13 and DDX27 expression were selected for subsequent studies. Collectively, these findings suggest that circ_RNF13 and DDX27 were upregulated in CRC tumor samples and cells.

### 3.3. Knockdown of Circ_RNF13 Suppresses Stemness and Increases Chemosensitivity in CRC Cells

**Figure 3:**
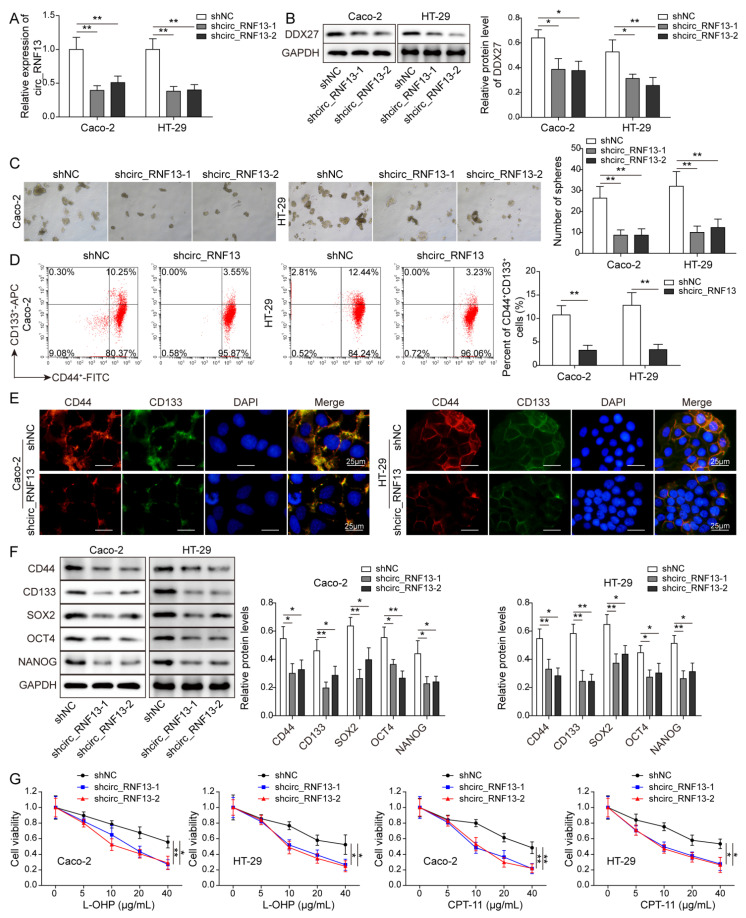

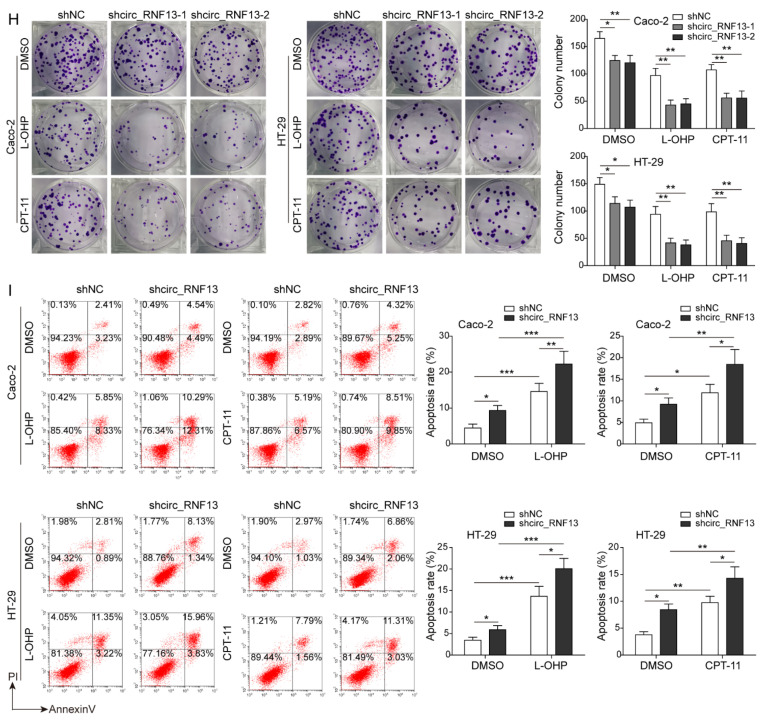
Knockdown of circ_RNF13 inhibits stemness and increases chemosensitivity in CRC cells. (**A**) The circ_RNF13 level in CRC cells was detected by qRT-PCR. (**B**) The protein level of DDX27 was detected by Western blot analysis. (**C**) The sphere-forming abilities of CRC cells were monitored by sphere formation assay. (**D**) The cell population of CD44^+^CD133^+^ cells was detected by flow cytometry. (**E**) The CD44 or CD133 level in CRC cells was detected by IF staining. Red, CD44; green, CD133; blue, DAPI. Scale bar, 25 µm. (**F**) The protein levels of stemness markers (CD44, CD133, SOX2, OCT4, and NANOG) were detected by Western blot analysis. (**G**) Cell viability was monitored by CCK-8 assay. (**H**) The colony-forming abilities of CRC cells were assessed by colony formation assay. (**I**) Cell apoptosis was measured by Annexin V-FITC/PI staining. Each experiment was performed at least three times independently. *, *p* < 0.05, **, *p* < 0.01, ***, *p* < 0.001. Full pictures of the Western blots are presented in Figure S2.

To delineate the biological roles of circ_RNF13 in CRC cells, loss-of function experiments were performed. Two shRNAs specific to circ_RNF13, namely sh-circ_RNF13-1 and sh-circ_RNF13-2, were employed to knockdown circ_RNF13 in HT-29 and Caco-2 cells. As expected, transfection of sh-circ_RNF13-1 or sh-circ_RNF13-2 successfully downregulated circ_RNF13 levels in both CRC cells (Figure 3A), along with the decrease in DDX27 (Figure 3B). Silencing of circ_RNF13 impaired the sphere-forming abilities of CRC cells (Figure 3C). Flow cytometry showed that silencing of circ_RNF13 decreased the number of CD44^+^CD133^+^ stem-like cells (Figure 3D). The expression of CD44 or CD133 was downregulated by circ_RNF13 silencing in CRC cells as detected by IF staining (Figure 3E). Western blot analysis further revealed that the depletion of circ_RNF13 reduced the expression of stemness markers, including CD44, CD133, SOX2, OCT4, and NANOG (Figure 3F). CCK-8 and colony formation assays showed that silencing of circ_RNF13 enhanced the sensitivities of CRC cells to L-OHP and CPT-11 (Figure 3G,H). In accordance with these results, flow cytometry revealed that cell apoptosis increased in circ_RNF13-knockdown cells upon L-OHP or CPT-11 treatment (Figure 3I). These findings suggest that knockdown of circ_RNF13 inhibited stemness and increased chemosensitivity in CRC cells.

### 3.4. Knockdown of DDX27 Inhibits Stemness and Increases Chemosensitivity in CRC Cells

**Figure 4:**
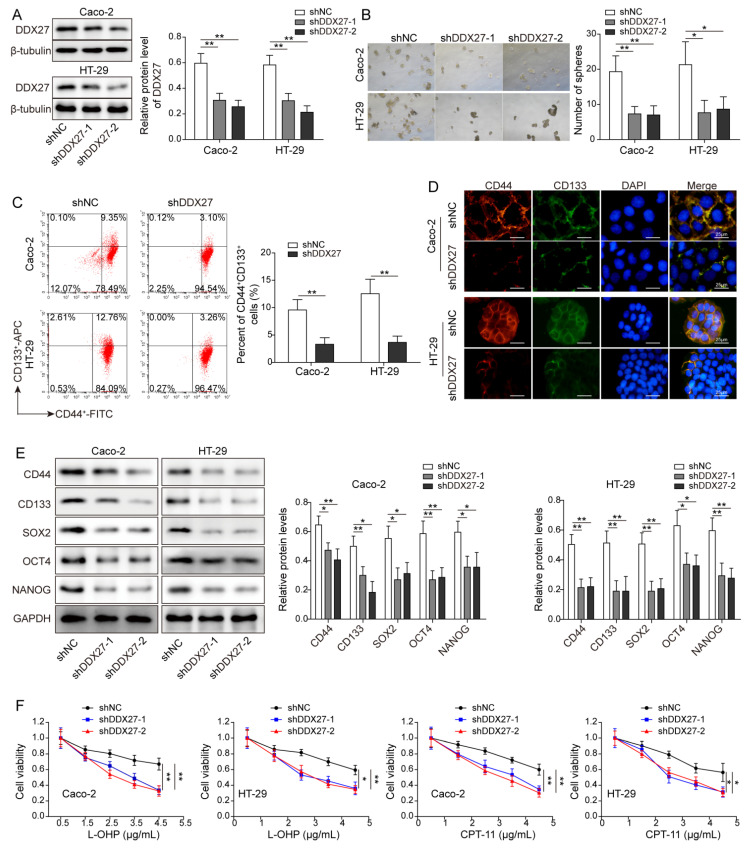

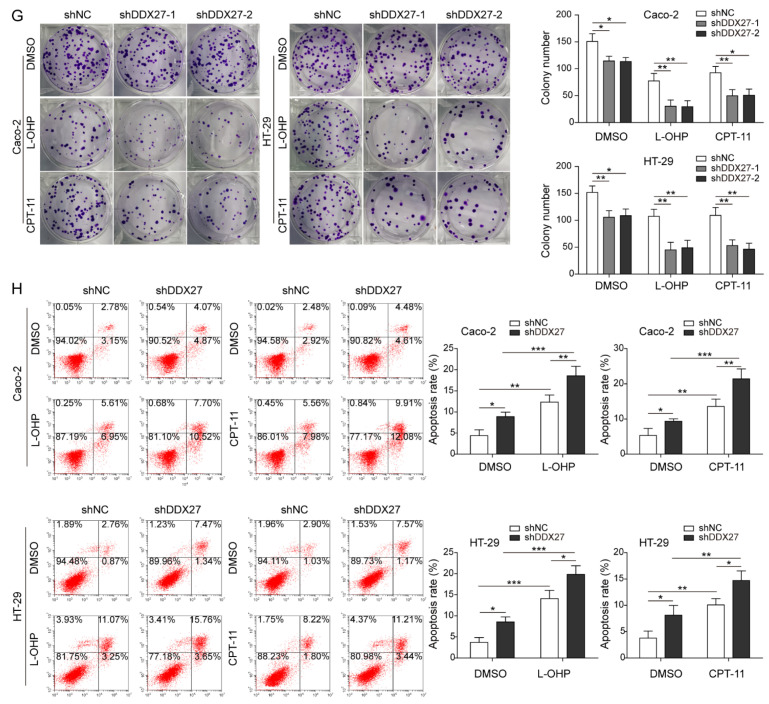
Knockdown of DDX27 inhibits stemness and increases chemosensitivity in CRC cells. (**A**) The protein level of DDX27 was detected by Western blot analysis. (**B**) The sphere-forming abilities of CRC cells were monitored by sphere formation assay. (**C**) The cell population of CD44^+^CD133^+^ cells was detected by flow cytometry. (**D**) The CD44 or CD133 level in CRC cells was detected by IF staining. Red, CD44; green, CD133; blue, DAPI. Scale bar, 25 µm. (**E**) The protein levels of stemness markers (CD44, CD133, SOX2, OCT4, NANOG) were detected by Western blot analysis. (**F**) Cell viability was monitored by CCK-8 assay. (**G**) The colony-forming abilities of CRC cells were assessed by colony formation assay. (**H**) Cell apoptosis was measured by Annexin V-FITC/PI staining. Each experiment was performed at least three times independently. *, *p* < 0.05, **, *p* < 0.01, ***, *p* < 0.001. Full pictures of the Western blots are presented in Figure S2.

We next sought to investigate the role of DDX27 in CRC cells. Compared with sh-NC, transfection of sh-DDX27-1 or sh-DDX27-2 caused a significant reduction in DDX27 in both HT-29 and Caco-2 cells as detected by Western blot analysis (Figure 4A). Similarly, a lack of DDX-27 suppressed sphere formation in CRC cells (Figure 4B) was detected. The percentage of CD44^+^CD133^+^ cells decreased in CRC cells (Figure 4C), and the CD44 or CD133 level was downregulated by DDX27 silencing (Figure 4D). Moreover, the protein levels of CD44, CD133, SOX2, OCT4, and NANOG were reduced in DDX27-knockdown cells (Figure 4E). Furthermore, functional experiments revealed that the knockdown of DDX27 enhanced L-OPH- or CPT-11-induced cytotoxicity (Figure 4F), impairment of colony formation (Figure 4G), and enhancement of cell apoptosis (Figure 4H). Collectively, these findings indicate that the effects of DDX27 silencing on stemness and chemosensitivity were similar to circ_RNF13 knockdown in CRC cells. 

### 3.5. Circ_RNF13 Regulates DDX27 Expression via TRIM24-Mediated Transcriptional Regulation

**Figure 5:**
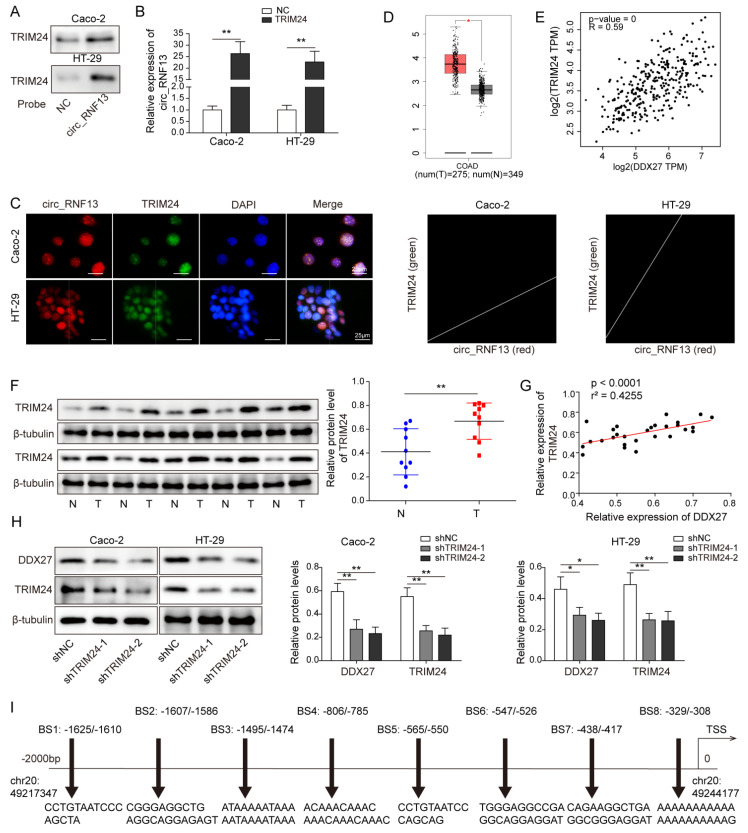

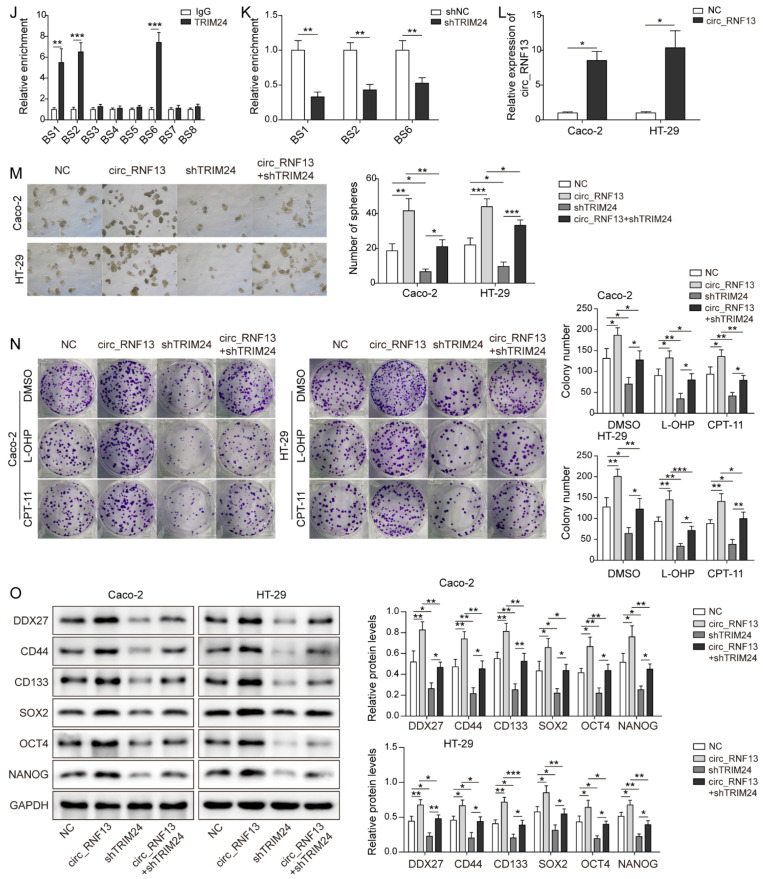
Circ_RNF13 regulates DDX27 expression via TRIM24-mediated transcriptional regulation. (**A**) The association between circ_RNF13 and TRIM24 was determined by RNA pull-down assay. Scrambled RNA served as a negative control. (**B**) The interaction between circ_RNF13 and TRIM24 was detected by RIP assay. (**C**) The subcellular localizations of TRIM24 and circ_RNF13 were detected by FISH/IF staining. Red, circ_RNF13; green, TRIM24; blue, DAPI. Scale bar, 25 µm. (**D**) Analysis of TRIM24 expression in CRC tumor samples based on the TCGA database. (**E**) The correlation between TRIM24 and DDX27 in CRC tumor samples based on the TCGA database. (**F**) The protein level of TRIM24 was detected by Western blot analysis in CRC tumor samples. (**G**) The correlation between TRIM24 and DDX27 in CRC tumor samples. (**H**) The protein levels of DDX27 and TRIM24 were detected by Western blot analysis in CRC cells. (**I**) Putative binding sites of TRIM24 on DDX27 promoter. (**J**,**K**) The interaction between TRIM24 and the DDX27 promoter was detected by ChIP assay. (**L**) The circ_RNF13 level in CRC cells was detected by qRT-PCR. (**M**) The sphere-forming abilities of CRC cells were monitored by sphere formation assay. (**N**) The colony-forming abilities of CRC cells were assessed by colony formation assay. (**O**) The protein levels of DDX27 and stemness markers (CD44, CD133, SOX2, OCT4, and NANOG) were detected by Western blot analysis. Each experiment was performed at least three times independently. *, *p* < 0.05, **, *p* < 0.01, ***, *p* < 0.001. Full pictures of the Western blots are presented in Figure S2.

In order to unravel the underlying mechanism by which the circ_RNF13/DDX27 axis regulates stemness and chemosensitivity, we further tested the association between circ_RNF13 and DDX27 in CRC cells. The transcription factors of DDX27 were predicted by AnimalTFDB, and the top six transcription factors with high reliability were screened, including ZNF384, GTF3C2, IRF5, GLYR1, POLR3A, and TRIM24. Among these transcription factors, RPIseq showed that the random forest (RF) and support vector machine (SVM) of TRIM24 were 0.75 and 1, respectively (Figure S1A). These data indicate the high reliability of TRIM24, and it was thus selected for subsequent validation. Consistent with the result of the bioinformatics analysis using RPISeq, the RNA pull-down assay revealed that biotinylated circ_RNF13 successfully pulled down TRIM24 in CRC cells (Figure 5A). Conversely, an antibody against TRIM24 also enriched circ_RNF13 as detected by the RIP assay (Figure 5B). FISH coupled with IF staining further illustrated the co-localization of circ_RNF13 and TRIM24 in CRC cells (Figure 5C), indicating that TRIM24 might act as an interacting molecule of circ_RNF13 in CRC. Interestingly, TCGA data showed that TRIM24 was upregulated by 1.75-fold in CRC tumor samples (Figure 5D), and there was a positive correlation between TRIM24 and DDX27 in CRC tumor samples (Figure 5E). Consistently, Western blot analysis showed that TRIM24 was elevated by 1.62-fold in CRC tumor samples (n = 10), compared with their normal counterparts (Figure 5F). The positive correlation between TRIM24 and DDX27 was also found in collected CRC tumor samples (Figure 5G). In addition, silencing of TRIM24 resulted in a decrease in DDX27 in both HT-29 and Caco-2 cells (Figure 5H). Bioinformatics analysis predicted the putative binding sites (BSs) of TRIM24 on the DDX27 promoter. To determine the BSs involved in TRIM24-mediated regulation, eight putative BSs were designated and are shown in Figure 5I. As shown in Figure 5J, the ChIP assay with an anti-TRIM24 antibody showed the enrichment of the DDX27 promoter fragments, including fragments containing BS1, BS2, and BS6. Silencing of TRIM24 remarkably reduced the anti-TRIM24 antibody-mediated enrichment on the DDX27 promoter (Figure 5K). The biological roles of the circ_RNF13/TRIM24/DDX27 axis were further investigated by functional experiments. As presented in Figure 5L, transfection of the circ_RNF13 overexpression construct caused a significant induction of circ_RNF13 in both HT-29 and Caco-2 cells. Overexpression of circ_RNF13 enhanced sphere formation, whereas TRIM24 silencing exerted an opposite effect. Moreover, the circ_RNF13-induced sphere formation was attenuated by TRIM24 knockdown (Figure 5M). The colony formation assay revealed that circ_RNF13 overexpression induced chemoresistance to L-OHP or CPT11, while knockdown of TRIM24 enhanced chemosensitivity and abrogated circ_RNF13-induced chemoresistance in CRC cells (Figure 5N). Furthermore, Western blot analysis showed that circ_RNF13 positively regulated the expression of DDX27 and stemness markers, whereas these proteins were downregulated in TRIM24-knockdown cells. Silencing of TRIM24 also reversed circ_RNF13-mediated upregulation of DDX27 and stemness markers (Figure 5O). Taken together, these findings indicate that circ_RNF13 regulated DDX27 expression via TRIM24-mediated transcriptional regulation.

### 3.6. Circ_RNF13 Stabilizes TRIM24 via Suppressing FBXW7-Mediated TRIM24 Degradation

**Figure 6:**
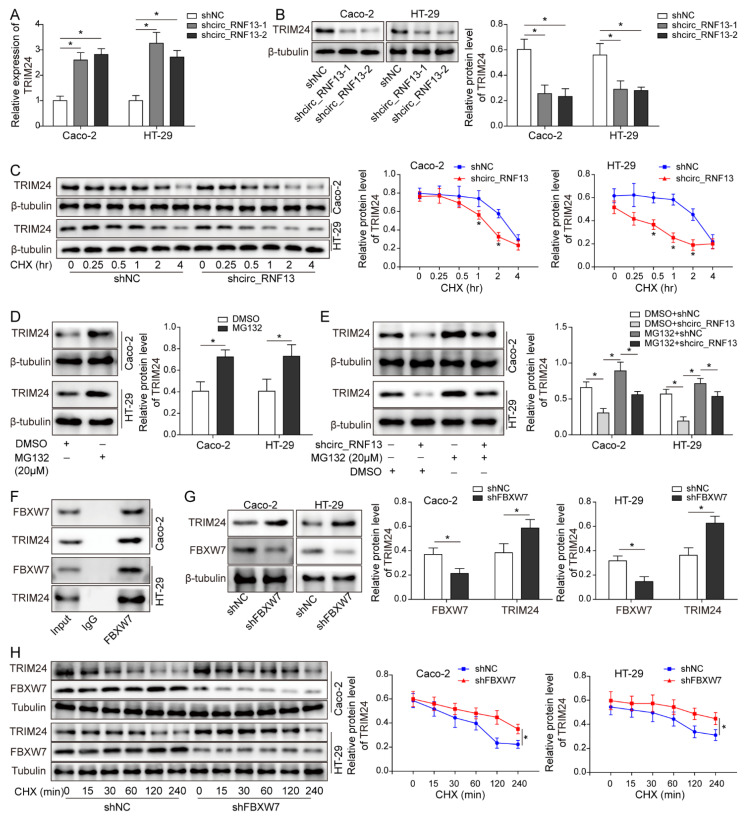

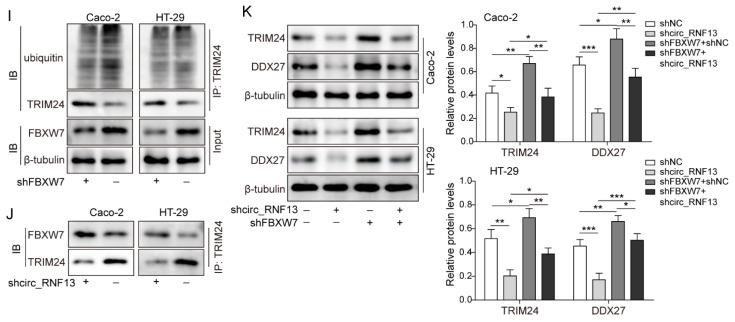
Circ_RNF13 stabilizes TRIM24 via suppressing FBXW7-mediated TRIM24 degradation. (**A**) The mRNA level of TRIM24 was detected by qRT-PCR. (**B**) The protein level of TRIM24 was detected by Western blot analysis. (**C**) The transfected HT-29 and Caco-2 cells were treated with CHX. The protein level of TRIM24 was detected by Western blot analysis. (**D**) HT-29 and Caco-2 cells were treated with MG132. The protein level of TRIM24 was detected by Western blot analysis. (**E**) The shcirc_RNF13-transfected HT-29 and Caco-2 cells were treated with MG132. The protein level of TRIM24 was detected by Western blot analysis. (**F**) The direct interaction between FBXW7 and TRIM24 was detected by Co-IP. (**G**) HT-29 and Caco-2 cells were transfected with sh-FBXW7. The protein levels of TRIM24 and FBXW7 were detected by Western blot analysis. (**H**) The transfected HT-29 and Caco-2 cells were treated with CHX. The protein levels of TRIM24 and FBXW7 were detected by Western blot analysis. (**I**) The ubiquitination of TRIM24 was detected by Co-IP. (**J**) HT-29 and Caco-2 cells were transfected with sh-circ_RNF13. The interaction between FBXW7 and TRIM24 was detected by Co-IP. (**K**) HT-29 and Caco-2 cells were transfected with sh-circ_RNF13 or/and sh-FBXW7. The protein levels of DDX27 and TRIM24 were detected by Western blot analysis. Each experiment was performed at least three times independently. *, *p* < 0.05, **, *p* < 0.01, ***, *p* < 0.001. Full pictures of the Western blots are presented in Figure S2.

To further delineate the mechanism by which circ_RNF13 regulated TRIM24, loss-of function experiments were performed. As presented in Figure 6A, knockdown of circ_RNF13 upregulated TRIM24 mRNA in CRC cells. By contrast, Western blot analysis showed that silencing of circ_RNF13 led to a remarkable decrease in TRIM24 at the protein level (Figure 6B). We thus speculated that circ_RNF13 might regulate TRIM24 protein expression via post-translational regulation. To test this hypothesis, TRIM24 stability was examined in the presence of protein synthesis inhibitor cycloheximide (CHX). In CRC cells, silencing of circ_RNF13 accelerated the degradation of TRIM24 (Figure 6C). In addition, the degradation of TRIM24 was blocked by the proteasome inhibitor MG132 (Figure 6D), indicating the involvement of the ubiquitin–proteasome pathway in TRIM24 degradation in CRC cells. MG132, at least in part, alleviated sh-circ_RNF13-accelerated TRIM24 degradation (Figure 6E). Bioinformatics analysis based on UbiBrowser predicted eight putative E3 ubiquitin ligases responsible for TRIM24 degradation, including MDM2, FBXW7, TRIM25, NEDD4, TRIM11, RNF180, SYVN1, and MIB1 (Figure S1B). Western blot analysis showed that knockdown of FBXW7 increased TRIM24 expression in Caco-2 and HT-29 cells, while silencing of the other E3 ligases had no significant effect on TRIM24 protein level (Figure S1C), suggesting that FBXW7 might be implicated in the regulation of TRIM24 degradation. Consistently, Co-IP revealed that FBXW7 and TRIM24 were components of the same protein complex in CRC cells (Figure 6F). Moreover, knockdown of FBXW7 caused a significant decrease in FBXW7, along with an increase in TRIM24 in CRC cells (Figure 6G). Silencing of FBXW7 slowed TRIM24 degradation in CRC cells (Figure 6H), suggesting the critical role of FBXW7 in TRIM24 degradation. Co-IP further revealed that a lack of FBXW7 suppressed the ubiquitination of TRIM24 (Figure 6I). Furthermore, an increased association between FBXW7 and TRIM24 was observed in circ_RNF13-knockdown cells (Figure 6J). Western blot analysis showed that knockdown of circ_RNF13 led to a reduction in TRIM24 or DDX27. On the contrary, silencing of FBXW7 exerted an opposite effect on TRIM24 and DDX27 levels, and FBXW7 knockdown also partially abrogated the effects of sh-circ_RNF13 in CRC cells (Figure 6K). Collectively, these findings suggest that circ_RNF13 stabilized TRIM24 via suppressing FBXW7-mediated TRIM24 degradation.

### 3.7. Knockdown of Circ_RNF13 Impairs Stemness and Enhances Chemosensitivity of CRC In Vivo

**Figure 7:**
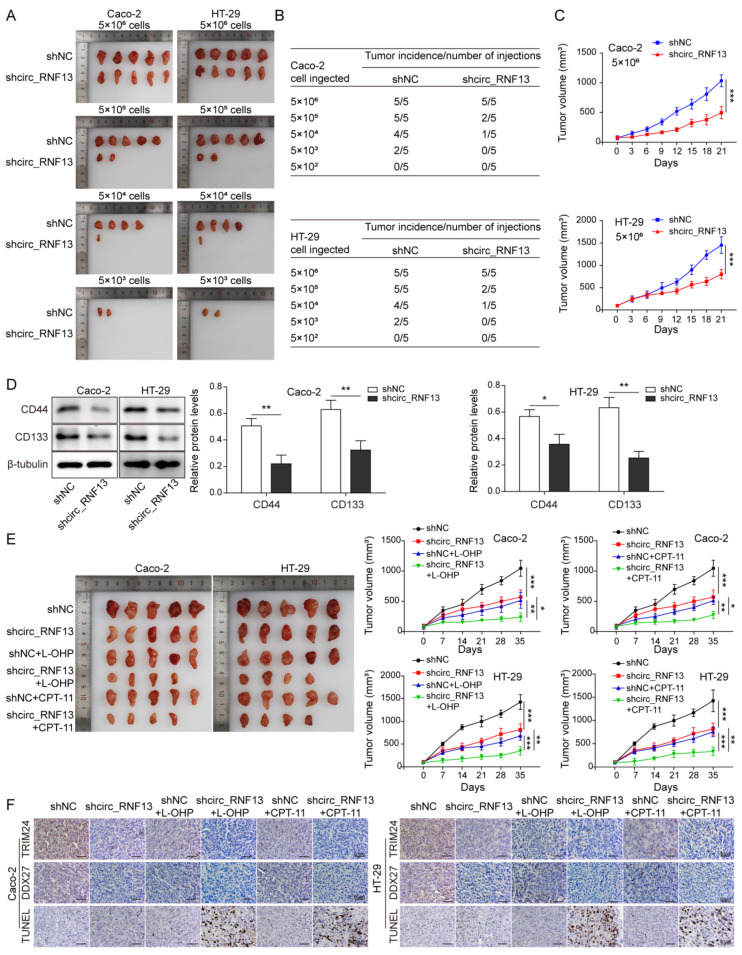
Knockdown of circ_RNF13 impairs the stemness and enhances the chemosensitivity of CRC in vivo. (**A**) Photographs of xenograft tumors. (**B**) The association between cell density and tumor incidence. (**C**) Quantitative analysis of tumor volume. (**D**) The protein levels of CD44 and CD133 in xenograft tumors were detected by Western blot analysis. (**E**) Photographs of xenograft tumors upon L-OHP or CPT-11 treatment. Quantitative analysis of tumor volume. (**F**) The immunoreactivity of TRIM24 and DDX27 in xenograft tumors were assessed by IHC analysis. Cell apoptosis in xenograft tumors was detected by TUNEL assay. Scale bar, 50 µm. Each experiment was performed at least three times independently. *, *p* < 0.05, **, *p* < 0.01, ***, *p* < 0.001. Full pictures of the Western blots are presented in Figure S2.

In order to validate the in vitro findings in the mouse model, a xenograft study was conducted. 5 × 10^6^, 5 × 10^5^, 5 × 10^4^, 5 × 10^3^, and 5 × 10^2^ transfected HT-29 and Caco-2 cells were injected into nude mice. As presented in Figure 7A–C, silencing of circ_RNF13 decreased the tumor size, volume, and incidence, compared with the control group. It is worth noting that the cell density was associated with tumor incidence (Figure 7A–C). Western blot analysis further showed that circ_RNF13 knockdown decreased the expression of stemness marker CD44 and CD133 in xenograft tumors (Figure 7D), indicating that a lack of circ_RNF13 impaired the stemness of CRC in vivo. Subsequent experiments revealed that silencing of circ_RNF13 enhanced the chemosensitivity to L-OHP or CPT-11 in which the tumor size and volume were notably reduced in sh-circ_RNF13+L-OHP or sh-circ_RNF13+CPT-11 groups, compared with the corresponding controls (Figure 7E). In addition, IHC showed that the immunoreactivity of TRIM24 or DDX27 in xenograft tumors was downregulated by circ_RNF13 knockdown, and the combination of sh-circ_RNF13 with L-OHP or CPT-11 treatment caused a lower expression of TRIM24 or DDX27 (Figure 7F). circ_RNF13 knockdown or L-OHP/CPT-11 treatment triggered cell apoptosis in xenograft tumors, and cell apoptosis was further potentiated in the sh-circ_RNF13+L-OHP or sh-circ_RNF13+CPT-11 groups (Figure 7F). These data indicate that knockdown of circ_RNF13 impaired the stemness and enhanced the chemosensitivity of CRC in vivo.

## 4. Discussion

Emerging evidence indicates that circ_RNF13 exerted an oncogenic or tumor-suppressive role, depending on the cancer type. On one hand, circ_RNF13 promotes AML progression via targeting miR-1224-5p [14], and it also promotes the malignant progression of PC via the miR-139-5p/IGF1R axis [15]. On the other hand, circ_RNF13 suppresses metastasis of LAD by sponging miR-93-5p [13]. Circ_RNF13 also regulates glycolysis in NPC via modulating the SUMO2/GLUT1 axis, thereby inhibiting NPC proliferation and metastasis [16]. It is well-established that CSCs play critical roles in recurrence, metastasis, and chemoresistance [24,25]. In recent years, circRNAs have emerged as novel players in stemness and chemoresistance in various cancers [26]. Previous studies have mainly focused on the circ_RNF13-miRNA networks; however, the effects of circ_RNF13 on stemness and chemoresistance are largely uninvestigated. In this study, our findings illustrated the mechanism by which circ_RNF13 regulated the stemness and chemoresistance of CRC by transcriptional regulation of DDX27 mediated by TRIM24 stabilization, thereby broadening the understanding of the oncogenic role of circ_RNF13.

Circ_RNF13 was derived from back-spliced exons 2-8 of the RNF13 gene. It was more stable than linear RNF13 which was attributed to the lack of 5′ to 3′ polarity and poly(A) tails. We demonstrated that circ_RNF13 was elevated in CRC tumor samples and cells, which was in line with the circRNA expression profile [17]. CRC patients with high circ_RNF13 levels are associated with poor OS, and it was positively correlated with several clinical parameters, suggesting that circ_RNF13 might be a potential prognostic marker in CRC. CSCs play an indispensable role in the therapeutic failure of CRC [27]. The sphere formation assay coupled with Western blot analysis and IF staining showed decreased sphere formation activity and reduced expression of stemness markers in circ_RNF13-knockdown cells. CSC overpopulation contributes to colon tumorigenesis [28]. Flow cytometry revealed that silencing of circ_RNF13 also decreased the CSC population. This was accompanied by an increased sensitivity to chemotherapy. As expected, CCK-8, colony formation, and cell apoptosis assays unequivocally showed that the circ_RNF13-knockdown cells were more sensitive to L-OHP or CPT-11 treatment. These findings were also validated in vivo. Similarly, our data illustrated that DDX27 was markedly elevated in CRC tumor samples and cells which was in accordance with previous reports [8,9]. It has been illustrated that DDX27 promotes cell proliferation, migration, and invasion, but it inhibits apoptosis via activating NF-κB signaling in CRC [8]. Moreover, the aberrant expression of DDX27 is associated with the stem-like activity of CRC cells [9]. In line with this study, we showed that knockdown of DDX27 suppressed stemness and increased chemosensitivity in CRC cells. These findings suggest the pivotal roles of circ_RNF13 and DDX27 in CRC stemness and chemosensitivity. TRIM24 is elevated in CRC, and TRIM24 levels are negatively correlated with OS of CRC patients [20,29]. Mechanistical studies have revealed that the DANCR/KAT6A complex enhances the interaction between TRIM24 and H3K23ac, thereby activating YAP transcription in CRC [29]. Consistently, we also showed that TRIM24 was highly expressed in CRC tissue. More importantly, RNA pull-down, RIP assays, and FISH/IF staining confirmed the association between circ_RNF13 and TRIM24, indicating that TRIM24 might be a downstream molecule of circ_RNF13 in CRC cells. Additionally, TRIM24 was identified as a transcription factor that regulated DDX27 expression. Collectively, our data suggested that circ_RNF13 interacted with TRIM24, thus promoting TRIM24-mediated transcriptional regulation of DDX27 and ultimately modulated stemness and chemosensitivity in CRC. Furthermore, the discrepancy of mRNA and protein levels of TRIM24 in circ_RNF13-knockdown cells promoted us to speculate that circ_RNF13 might regulated TRIM24 levels via post-translational regulation. E1 ubiquitin-activating enzyme, E2 ubiquitin-activating enzyme, and E3 ubiquitin ligase are well-known players in the ubiquitin–proteasome pathway [30]. FBXW7, an E3 ubiquitin ligase, attracted our attention due to its downregulation in CRC [31,32], as well as the bioinformatics analysis which predicted the direct association between FBXW7 and TRIM24. Interestingly, the knockdown study coupled with Co-IP revealed that FBXW7 mediated the degradation of TRIM24 via the ubiquitin–proteasome pathway, and circ_RNF13 stabilized TRIM24 via suppressing FBXW7-mediated degradation. Double knockdown of FBXW7 and circ_RNF13 partially rescued the reduction of TRIM24 and DDX27. Partial rescue effects by double knockdown have also been reported in previous studies [33,34]. Our findings suggest that FBXW7 might not be the only downstream effector of the circ_RNF13/TRIM24/DDX27 axis. However, this regulatory mechanism needs to be validated in vivo in future studies, and the crosstalk with other mechanisms also merits further investigation. It is worth noting that the direct association between circ_RNF13 and TRIM24 blocked the interaction between FBXW7 and TRIM24, thereby inhibiting FBXW7-mediated degradation of TRIM24. Similar mechanisms have been reported in neuroblastoma and hepatocellular carcinoma [35,36]. For instance, circ-SORE blocks the binding between YBX1 and E3 ligase PRP19 by interacting with YBX1, thus suppressing the PRP19-mediated degradation of YBX1 [36]. In summary, circ_RNF13 regulated DDX27 expression via TRIM24-mediated transcriptional regulation, and it also stabilized TRIM24 via suppressing FBXW7-mediated degradation, thus modulating the stemness and chemosensitivity of CRC.

## 5. Conclusions

In conclusion, circ_RNF13 regulated the stemness and chemosensitivity of CRC by transcriptional regulation of DDX27 mediated by TRIM24 stabilization. These findings provided novel insights into chemoresistance in CRC, and the circ_RNF13/TRIM24/DDX27 axis might be a promising target for chemotherapy. The positive correlations between circ_RNF13 and the clinicopathological characteristics of CRC patients indicate that circ_RNF13 might function as a potential prognostic biomarker for CRC.

## Figures and Tables

**Table 1:** The primers used in the qRT-PCR.

Primer	Sequence 5′-3′
circ_RNF13 (divergent) sense	GTCCAGGATAGACATAGAGC
circ_RNF13 (divergent) anti-sense	GTGTAGACTTGTGTGGCTGA
circ_RNF13 (convergent) sense	GCTCTCCATAGGGATGCTCA
circ_RNF13(convergent) anti-sense	GCAGGGAGGTCATCAAATGT
RNF13 sense	CCTCCCTGCAAGATTTGGTT
RNF13 anti-sense	TGTATCCTGCTCGTGCA
DDX27 sense	AGCCCGTGGACTTGACATTG
DDX27 anti-sense	GCATCTTCCGCTCATCTTCTC
TRIM24 sense	AGCCACAAATGCCTAAGC
TRIM24 anti-sense	AGGATGAGGAGGAAGAACTG
U6 sense	CTCGCTTCGGCAGCACA
U6 anti-sense	AACGCTTCACGAATTTGCGT
GAPDH sense	CCAGGTGGTCTCCTCTGA
GAPDH anti-sense	GCTGTAGCCAAATCGTTGT

**Table 2:** Antibodies used in this study.

Antibody	Vendor	Catalog No.	Working Dilution
DDX27	Invitrogen	PA5-61421	WB (1:500); IHC (1:50)
CD44	Abcam	ab51037	WB (1:2000); IF (1:100)
CD133	Abcam	ab19898	WB (1:1000); IF (1:100)
SOX2	Abcam	ab97959	WB (1:1000)
OCT4	Abcam	ab19857	WB (1:1000)
NANOG	Abcam	ab109250	WB (1:2000)
CD133-APC	Invitrogen	17-1338-42	Flow (0.125 μg)/test
CD44-FITC	Invitrogen	11-0441-82	Flow (0.5 μg)/test
TRIM24	Abcam	ab70560	WB (1:1000); IP/RIP (2 μg)
FBXW7	Abcam	ab109617	WB (1:1000); IP (2 μg)
β-tubulin	Abcam	ab6046	WB (1:1000)
GAPDH	Abcam	ab8245	WB (1:2000)

Invitrogen, Carlsbad, CA, USA; Abcam, Cambridge, UK.

**Table 3:** Relationships between circ_RNF13 expression and the clinicopathological characteristics of CRC patients.

Variables	Cases	Expression		*p* Value	x^2^
		Low (n = 25)	High (n = 25)		
Gender					
Male		14	12	0.5713	0.3205
Female		11	13		
Age					
≤62 years		10	11	0.7745	0.08210
>62 years		15	14		
Tumor size					
<5 cm		8	16	0.0235 *	5.128
≥5 cm		17	9		
Distant metastasis					
Negative		19	9	0.0044 **	8.117
Positive		6	16		
Differentiation					
Poor		16	12	0.2545	1.299
Well to moderate		9	13		
Lymph node metastasis					
Negative		18	10	0.0227 *	5.195
Positive		7	15		
TNM stage					
I+II		20	11	0.0087 **	6.876

* *p* < 0.05, ** *p* < 0.01.

## Data Availability

All data generated or analyzed during this study are included in this article. The datasets used and/or analyzed during the current study are available from the corresponding author on reasonable request.

## Supplementary Materials

The following supporting information can be downloaded at: https://www.mdpi.com/article/10.3390/cancers14246218/s1, Figure S1. Bioinformatic analysis based on RPIseq, Figure S2. Original Western blot.

## Abbreviations

5-FU, 5-fluorouracil; AML, acute myeloid leukemia; BS, binding site; CHX, cycloheximide; circRNA, circular RNA; ChIP, chromatin immunoprecipitation; CPT-11, irinotecan; CRC, colorectal cancer; CSCs, cancer stem cells; DDX27, dead-box helicase 27; FBXW7, F-box and WD repeat domain-containing 7; FISH, fluorescence in situ hybridization; gDNA, genomic DNA; IF, immunofluorescence; LAD, lung adenocarcinoma; L-OHP, oxaliplatin; MOC, Manders’ overlap coefficient; NPC, nasopharyngeal carcinoma; OS, overall survival; PC, pancreatic cancer; RF, random forest; SVM, support vector machine; RIP, RNA immunoprecipitation; RNF13, RING finger protein 13; rRNA, ribosomal RNA; TCGA, The Cancer Genome Atlas; TRIM24, tripartite motif containing 24.
