# Supplementary material for: Circ_RNF13 Regulates the Stemness and Chemosensitivity of Colorectal Cancer by Transcriptional Regulation of DDX27 Mediated by TRIM24 Stabilization
Source: Cancers (Basel). 2022 Dec 16;14(24):6218. doi: 10.3390/cancers14246218 (PMC9776557; doi:10.3390/cancers14246218)
Supplement: Supplementary file 1 [file cancers-14-06218-s001.zip › cancers-2011572-supplementary.pdf]

Supplemental Materials

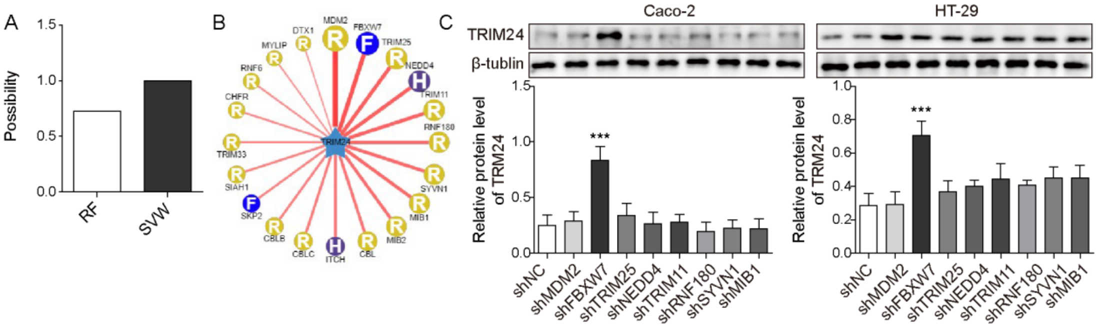

Figure S1. Bioinformatic analysis based on RPIseq. (A) The Random Forest (RF) and Support Vector Machine (SVM) of TRIM24 were determined by RPIseq. (B) The E3 ubiquitin ligase responsible for TRIM24 degradation was predicted using ubibrowser. (C) The protein level of TRIM24 was detected by western blot.

Fig2D

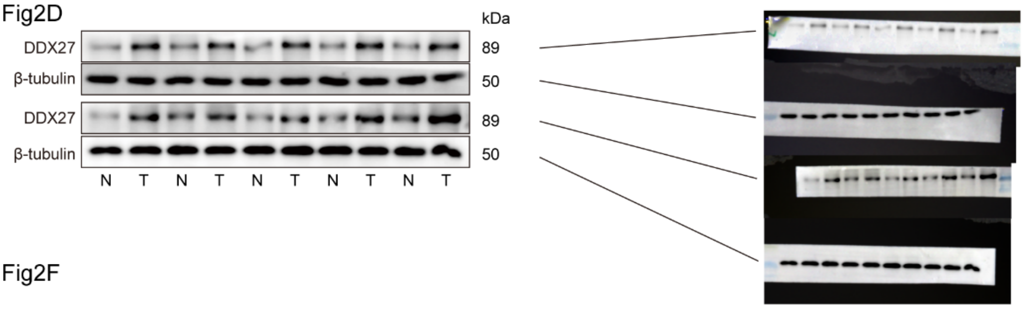

Fig2F

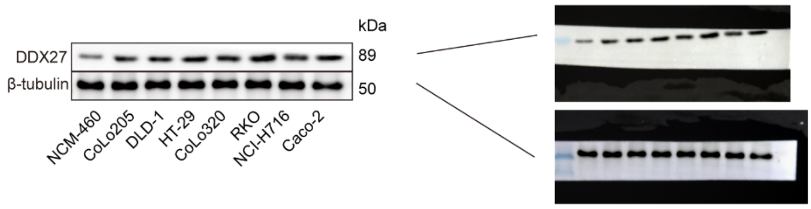

Fig3B

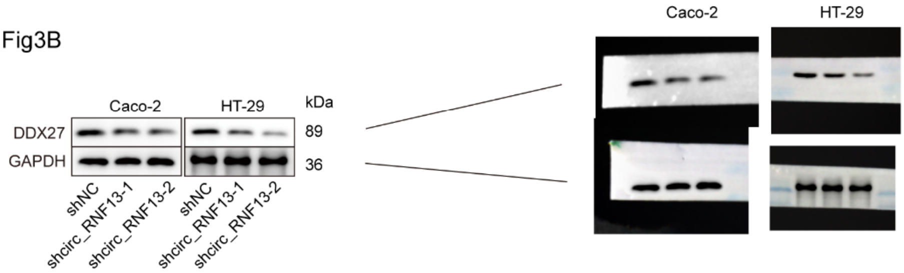

Fig3F

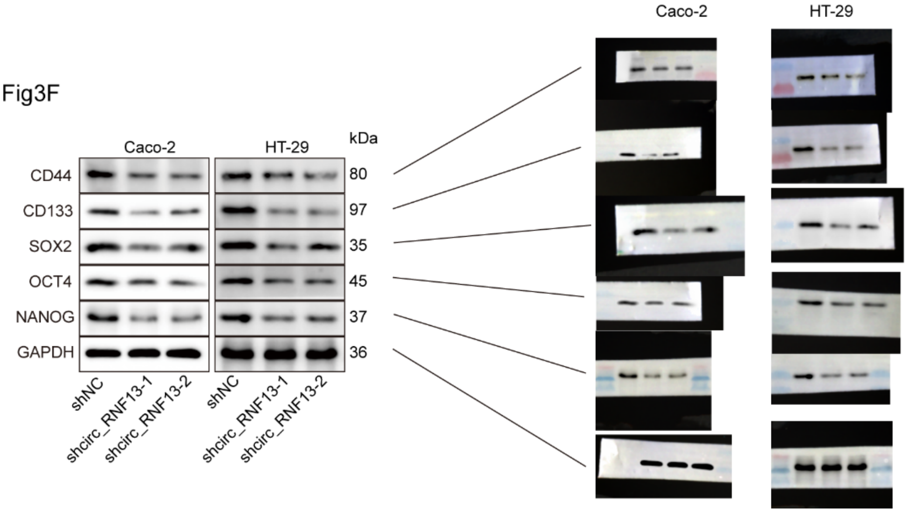

Fig4A

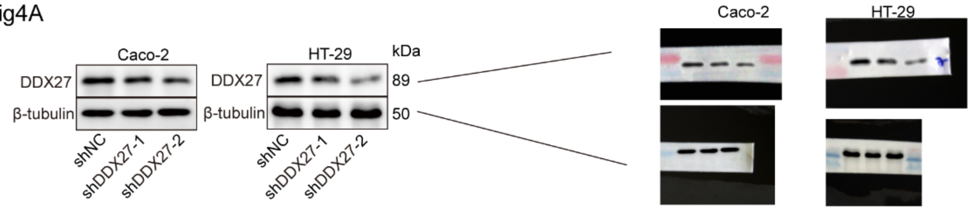

Fig4E

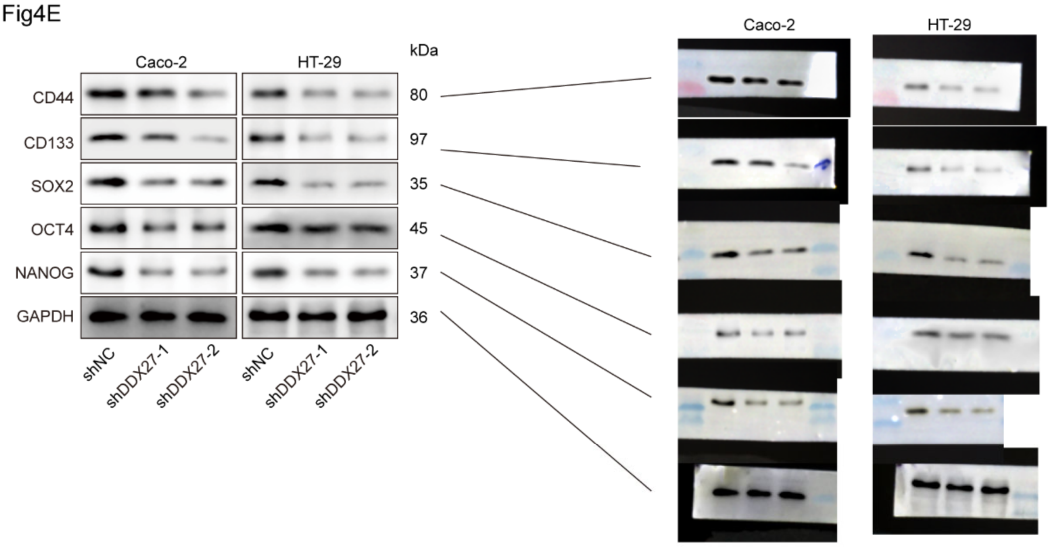

Fig5A

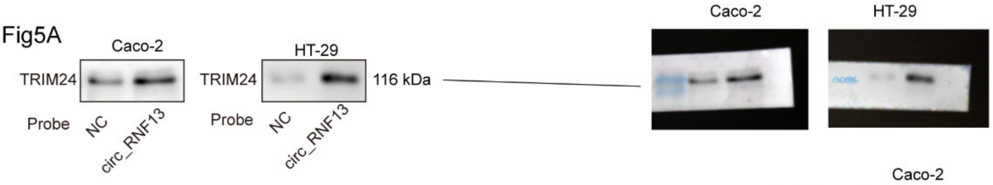

Fig5F

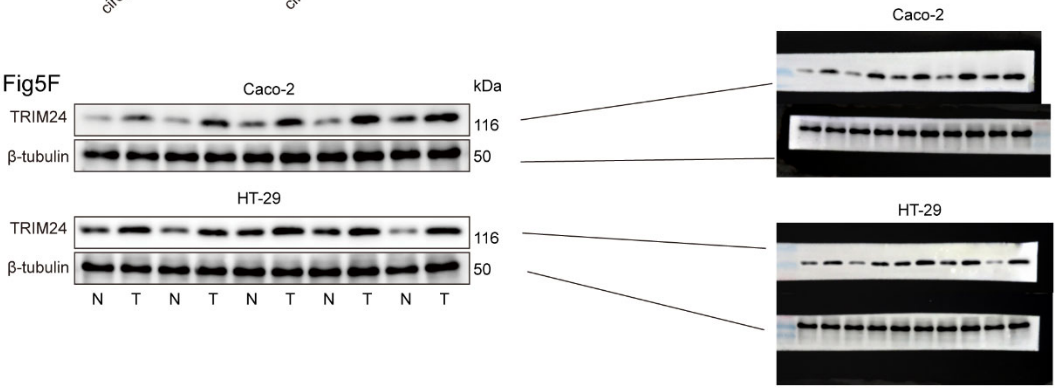

Fig5H

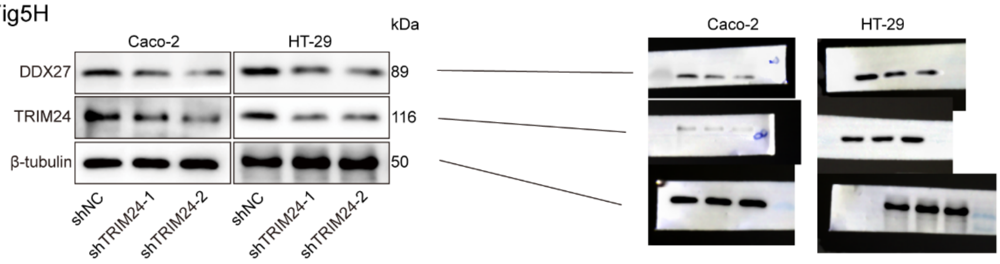

Fig5O

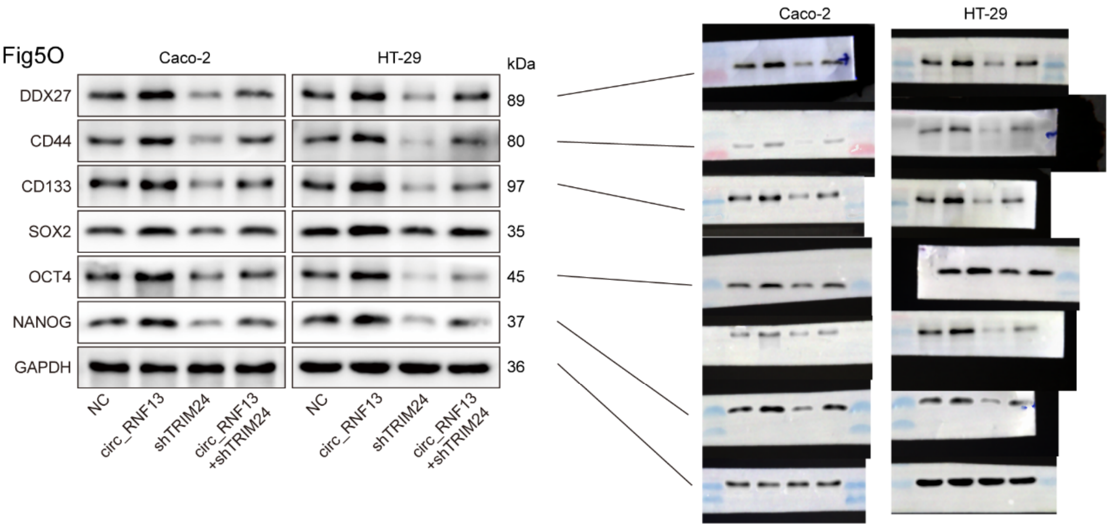

Fig6B

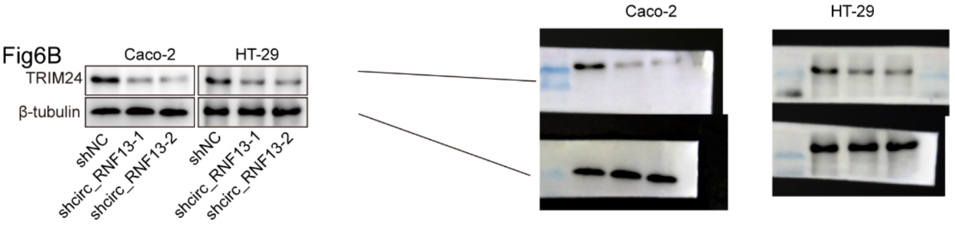

Fig6C

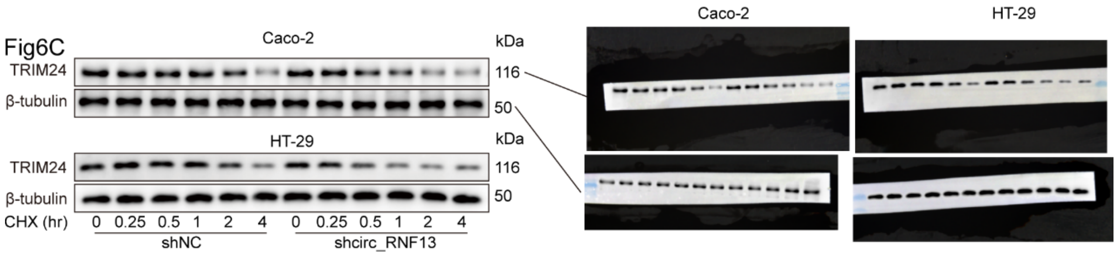

Fig6D

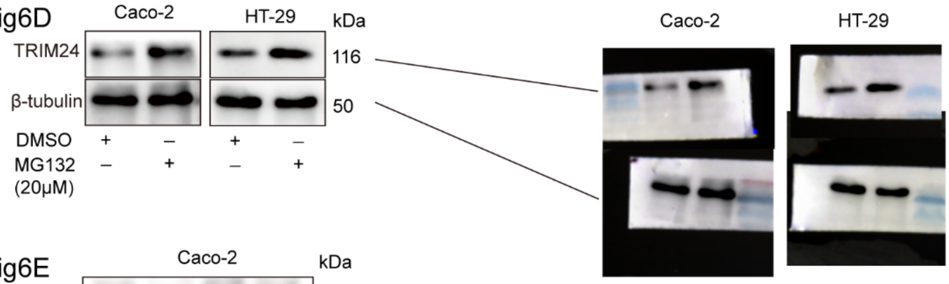

Fig6E

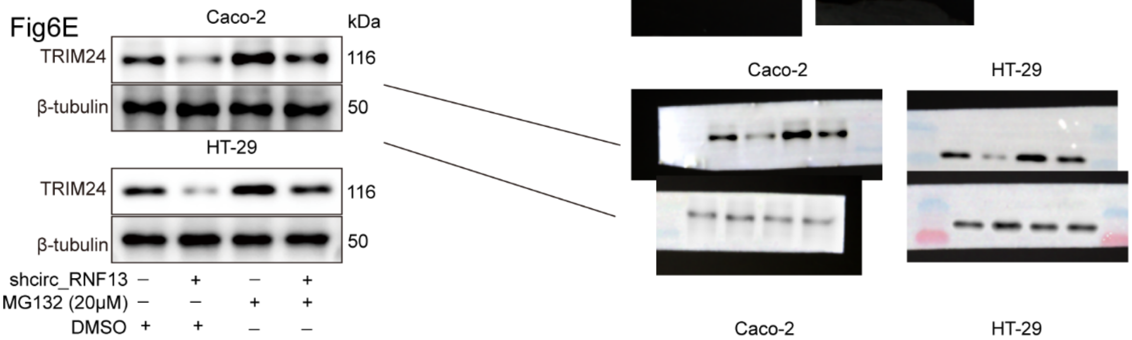

Fig6F

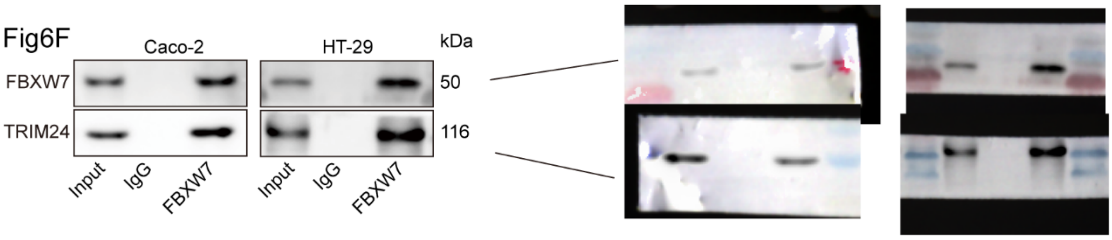

Fig6G

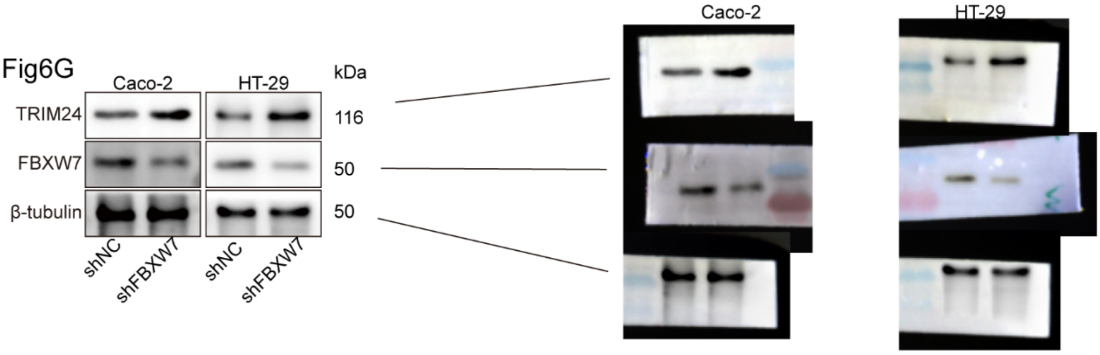

Fig6H

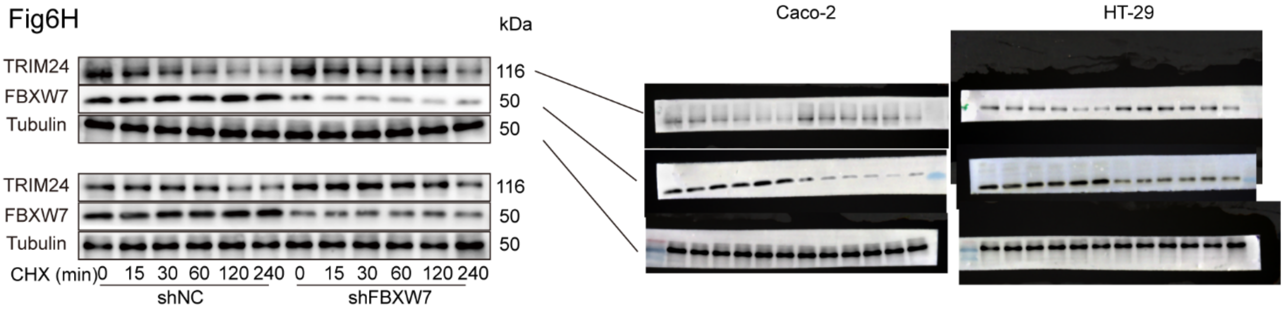

Fig6I

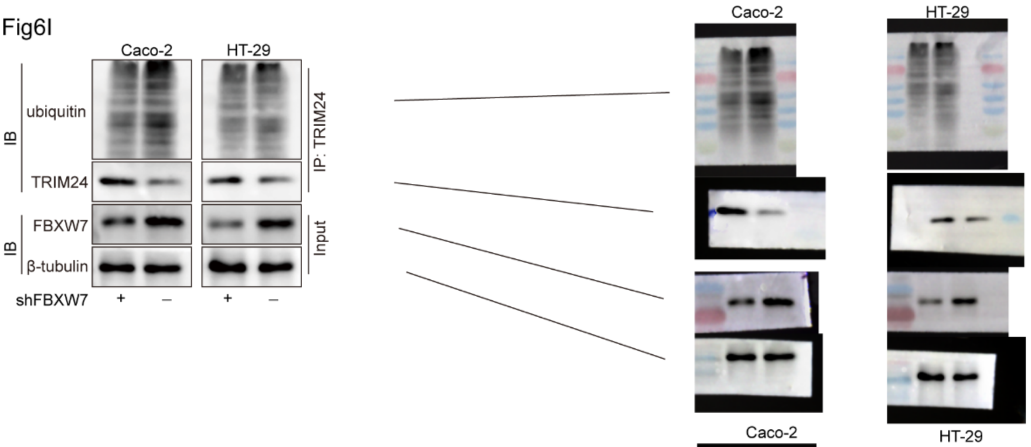

Fig6J

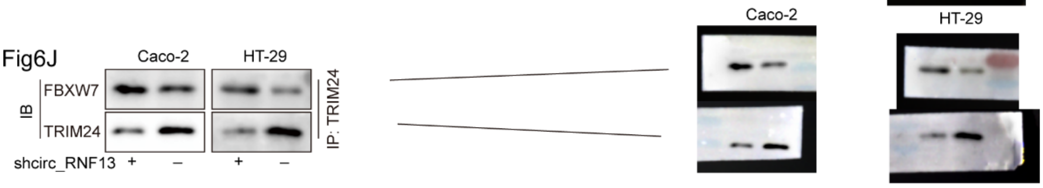

Fig6K

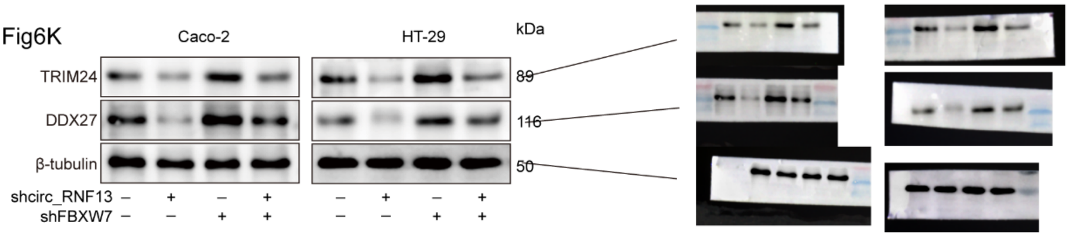

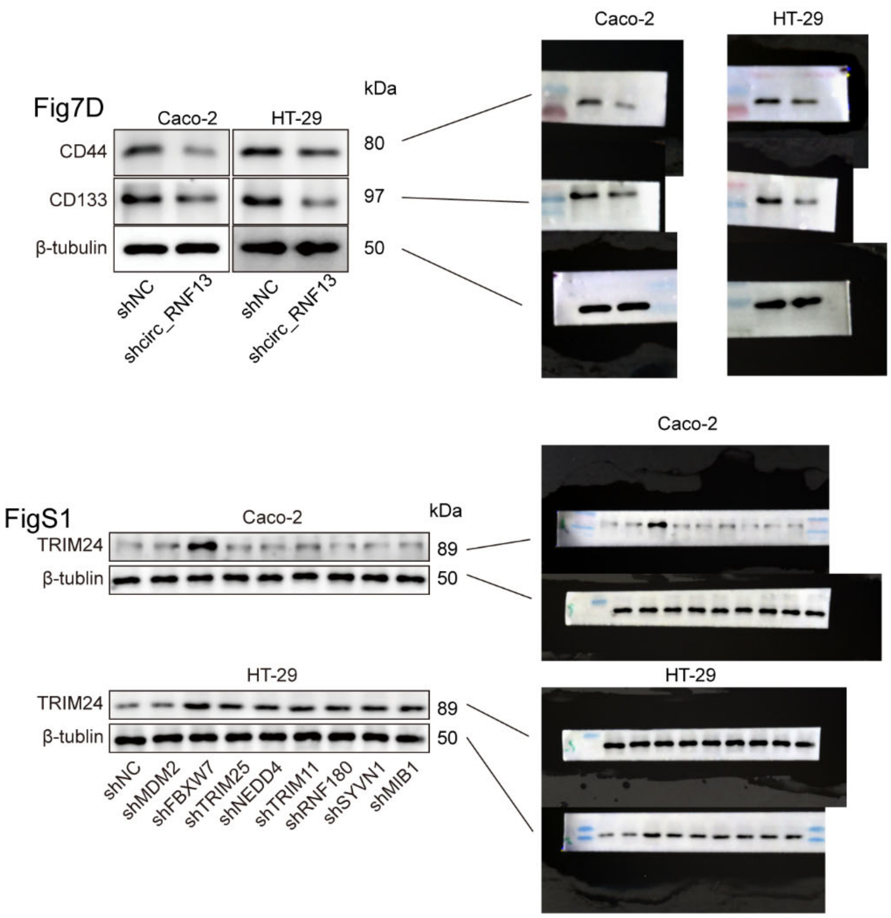

Figure S2. Original Western blot.
